# High sero-prevalence of hepatitis B virus and human immunodeficiency virus infections among pregnant women attending antenatal clinic at Temeke municipal health facilities, Dar es Salaam, Tanzania: a cross sectional study

**DOI:** 10.1186/s12884-017-1299-3

**Published:** 2017-04-07

**Authors:** Joel Manyahi, Yohannes Msigwa, Francis Mhimbira, Mtebe Majigo

**Affiliations:** 1 Department of Microbiology and Immunology, Muhimbili University of Health and Allied Sciences, Dar es Salaam, Tanzania; 2 Ifakara Health Institute, Dar es Salaam, Tanzania

**Keywords:** HBV, HBsAg, HIV, Pregnant women, Tanzania

## Abstract

**Background:**

Hepatitis B virus (HBV) and Human immunodeficiency virus (HIV) infection in pregnancy is associated with direct effect of pregnancy and potential viral transmission from mother to newborn. In Tanzania very little in known on prevalence of HBV infection and their associated factors among pregnant women in lower health facilities. The main objective of the study was to determine the prevalence of HBsAg, HIV and HBV-HIV co-infection among pregnant women attending antenatal clinics in Dar es Salaam.

**Methods:**

This cross sectional study was conducted in three Temeke municipal health-care facilities between May 2014 and June 2014. A total of 249 pregnant women attending antenatal clinic (ANC) were consecutively enrolled in the study. A data collection tool was used to extract socio-demographic characteristics from ANC card. Commercial ARCHITECT® ci4100™ was used to assess HBsAg status and liver function (Alanine amino-transferase (ALAT). HIV status was determined by anti-HIV antibody test.

**Results:**

Of 249 pregnant women enrolled the median age was 25 years (IQR 22–30) and most of them were married (72.4%). The overall prevalence of HBsAg and HIV were 8.03% (95% CI: 5.0–12.1%) and 17.2% (95% CI: 12.8–22.5%), respectively. HBV/HIV co-infection rate was 2.8% (95% CI; 1.3–5.4%). HBsAg positive rate was significantly high in women who were HIV positive (*p* < 0.05). Being employed /student were less associated with HBV infection (aOR 0.35, 95% CI 0.13–0.95). Only 3 (15%) of pregnant women with HBsAg positive had abnormal ALAT.

**Conclusions:**

High prevalence of HBV and HIV infections among pregnant women were reported in this setting thus calls for the national expansion of the integration of prevention of mother-to-child transmission (PMTCT) services for HBV infection.

## Background

Chronic hepatitis B virus and HIV infections remain a major global public health concern. Worldwide, about 350 million individuals are chronically HBV infected; half of them acquired infection either perinatal or in early childhood, majority found in endemic areas [1]. Annually, estimated more than 600,000 people die as a result of hepatitis B virus related liver diseases [2]. The risk of progression to chronic disease is inversely proportional to the age at which infection is acquired. Infection to women during pregnancy is associated with increased risks of transmission to a newborn [3, 4]. With impaired immunity in new-born, infection acquired during this stage may lead to further progression to liver cirrhosis and hepatocellular carcinoma later in life. HBV and HIV share the common mode of transmission and co- infection in pregnancy is associated with increased morbidity, mortality and untoward outcomes to a newborn. HIV co-infection increases HBV replication, leading to higher levels of detectable virus [5], and increases likelihood of perinatal transmission of HBV.

Prevention of mother to child transmission of hepatitis B virus is the most important strategy in the control of infection to newborn. Hepatitis B virus vaccine was introduced into the Tanzanian immunization and vaccine development program since2002 given at 4, 8, and 12 weeks as a DPT-HB [6]. However, the country has yet to introduce strategies to reduce the vertical transmission of HBV including screening for HBsAg, use of antivirals in the third trimester of pregnancy and at-birth prophylaxis of newborns which greatly reduces the risk of transmission [7, 8].

The prevalence of HBsAg in Tanzania varies considerably from 1.2 to 17.3% between different cohorts. Prevalence of 9.2 and 3.2% have been reported among HIV infected cohort in Southern rural and Northern Tanzania, respectively [9, 10]. At Muhimbili national hospital, in Dar es Salaam recent studies reported HBsAg prevalence of 1.2, 3.9, 8.8 and 17.3% among HIV children, pregnant women, blood donors and HIV adults, respectively [11–14]. These indicate that different cohort in different geographical locations at different level of health facilities have dissimilar prevalence of HBV infection. Very little in known on prevalence of Hepatitis B virus infection among pregnant women attending antenatal clinics at municipal hospital and health centers in Tanzania. Few studies reported previously were mostly conducted at referral and tertiary hospital in which population varies from district hospital and health centers.

World Health Organization (WHO) has called for integrated and standardized prevention of mother-to-child transmission (PMTCT) for HIV, syphilis and HBV [15]. Despite the call Tanzania has not scaled up PMTCT services for HBV infection. Understanding the epidemiology of Hepatitis B infections among pregnant women at different levels in Tanzania is of importance for optimizing HBV infection control strategies. Therefore, we conducted this study in Temeke municipality which has high annual growth rate in Tanzania, aiming at determining the prevalence of HBsAg and HIV among pregnant women attending antenatal clinics.

## Methods

### Study design and settings

This cross sectional study among pregnant women attending antenatal clinics (ANC) was conducted between May 2014 and June 2014. The study was carried out at 3 health-care facilities in Temeke municipal including Temeke hospital, Zakheem and Kizuiani health centers. Temeke Municipality is one of the three Municipalities within the Dar es Salaam City Council with a population of 1,368,881 and an estimated growth rate of 6.6% per year compared to national annual growth rate of 2.9%.

### Study population, sample size and sampling procedure

Participants were pregnant women attending ANC during the study period. The sample size was calculated using Kish and Lisle formula for cross sectional studies, *n* = z^2^ p(1-p)/d^2^. Where: z = Z score for 95% confidence interval = 1.96, p = prevalence, d = tolerable error =3%. A prevalence of 3.9% was used as p for a previous study in Tanzania [12]. Consequently, the minimal sample size of the study population was 159 participants. However, we consecutively recruited all pregnant women who were attending ANC clinic during the study period to increase the power of our study. Therefore a total of 249 pregnant women who met eligible criteria and consenting to participate in the study were recruited. Inclusion criteria was pregnant women aged > 18 years and women were excluded if knew their HBsAg status and if not residing in Temeke municipal.

### Data collection

Data were extracted from antenatal clinic card using data standard structured questionnaires. Information on social demographic characteristics including age, education levels, occupational status and marital status were recorded.

### Specimen collection, transport and storage

Five milliliters of blood were collected aseptically by veno-puncture into plain vacutainer tube. Half portion was used for HIV screening on site. Another half portion was packed into cool box then immediately delivered to central pathology laboratory at Muhimbili national hospital for further processing where sera were immediately separated after centrifugation.

### Laboratory procedures

#### Detection of hepatitis B surface antigen

Commercially available ARCHITECT® ci4100™ Integrated Immunoassay and Clinical Chemistry System (Abbott Laboratories, Abbott Park, IL, USA) was used to assess hepatitis B surface antigen (HBsAg) status. All specimens who were HBsAg positive, Alanine amino-transferase (ALT) were measured to assess liver function. The Architect C8000 automated analyzer was used to detect ALT levels. An Alanine amino-transferase value > 38 IU/L indicated abnormal liver function.

#### HIV screening

HIV infection among pregnant women was determined by anti-HIV antibody test (rapid test currently used in national algorithm for Tanzania). Alere Determine™ HIV1/2 (Alere Medical Co. Ltd. Matsudo-shi, Chiba-ken, Japan) was used for screening and negative results underwent no further testing. Positive samples were re-tested with Uni-Gold™ HIV (Trinity Biotech Manufacturing Ltd. Bray, Ireland). Specimens that were positive on both tests were considered HIV antibody positive. All discordant specimens were sent to Muhimbili national hospital for resolution. In addition few randomly selected specimens were sent to Muhimbili national hospital for quality assurance.

### Data analysis

Data were entered and analyzed using Microsoft excel 2010 and Stata version 11.2 respectively. Wilcox ranksum test and Fisher’s exact test were performed to examine the difference between categorical variables. A *p*-value of <0.05 was considered statistically significant. We determined the association between, the outcome HBsAg and associated factors using the univariate (crude odd ratio-cOR) and multivariate logistic regression (adjusted odd ratio-aOR) were performed to identify independent factors associated with HBsAg positivity. The point estimates are presented as odd ratio (OR) and their corresponding 95% Confidence Interval (95% CI).

## Results

### Study participants’ characteristics

A total of 249 pregnant women were enrolled in the study. Their age ranged from 18 to 40 years with median age of 25 years (IQR 22–30). The age group with highest proportion was < 24 years contributing 45.8% of all participants. Nearly-half (46.2%) of participants had secondary level of education and most participants were married 72.4%. Almost forty percent (39.8%) of the study participants were in second trimester and 52% were petty traders (Table 1).

**Table 1:** Socio-demographic characteristics of the study participants

Variables	Frequency (%)
Age, median (IQR)	25 (22–30)
Age groups	
< 24	114 (45.8)
25–30	86 (34.5)
> 31	49 (19.7)
Education status	
Primary	96 (38.6)
Secondary	115 (46.2)
College	38 (15.3)
Marital status	
Single	52 (20.9)
Married	182 (73.1)
Cohabiting	14 (5.6)
Divorced	1 (0.4)
Occupation status	
Employed	33 (13.3)
Petty traders	130 (52.2)
Unemployed	64 (25.7)
Student	22 (8.8)
HIV status	
Positive	43 (17.3)
Negative	206 (82.7)
Gestation age	
1^st^ trimester	80 (32.1)
2^nd^ trimester	99 (39.8)
3^rd^ trimester	70 (28.1)

### HBV and HIV infection

Of 249 pregnant women, the prevalence of HBsAg was 8.03% (95% CI: 5.0–12.1%). The HBsAg positive were simultaneously increasing with age, with women aged more than 35 years having the highest prevalence compared to other age groups. The HBsAg was significantly higher among women with primary school education (12.5% *p* < 0.05) (Table 2). Women who were married/cohabit and those who had no employment significantly had higher prevalence of HBsAg (*p* < 0.05). The gestation age was not statistically associated with HBsAg. The overall prevalence of HIV among this cohort was 17.2% (95% CI: 12.8–22.5). About 2.8% (95% CI; 1.3–5.4) of pregnant women were HBV and HIV co-infected. The rate of HBsAg positive was significantly higher in women who were HIV positive compared to HIV negative (*p* < 0.05). Of 20 pregnant women with HBsAg positive only 3 (15%) had abnormal elevated Alanine aminotransferase (ALAT).

**Table 2:** HBsAg results in relation to clinical and demographic characteristics

Variables	HBsAg positive *n* = 20	HBsAg negative *n* = 229	*p*-value
Age, median (IQR)	28 (23.5–31)	25 (22–29)	0.13*
Age groups			
< 24	8 (7.0)	106 (93.0)	
25–30	7 (8.14)	79 (91.9)	0.79
> 31	5 (10.2)	44 (89.8)	
Education status			
Primary	12 (12.5)	84 (87.5)	0.04
Secondary/College	8 (5.2)	145 (94.8)	
Marital status			
Single/divorced	1 (1.9)	52 (98.1)	0.047^#^
Married/Cohabit	19 (9.7)	177 (90.3)	
Occupation status			
Unemployed	10 (15.6)	54 (84.4)	0.01
Employed/student	10 (5.4)	175 (94.6)	
HIV status			
Positive	7 (16.3)	36 (83.7)	0.029
Negative	13 (6.3)	193 (93.7)	

*Wilcox ranksum test *p*-value; ^#^Fisher’s exact *p*-value

### Association of HBsAg and clinical-socio demographic characteristics

In a univariate analysis it was found that an increase in age was associated with increased odds of HBsAg positive (aOR 1.05 (0.97–1.14), though this association was not statistically significant (Table 3). Pregnant women who were HIV positive were nearly three times more likely to be HBsAg positive (cOR 2.89, 95% CI 1.08–7.73) than women who were HIV negative. Women who were married/cohabiting were also more likely to be HBsAg positive compared to those who were single/divorced (cOR 5.58, 95% CI 0 .73–42.69). Having secondary /college education (cOR 0.39, 95% CI 0.15–0.98) and being employed/student (cOR 0.31, 95% CI 0.12–0.78, *p* < 0.05) were significantly at reduced risk of Hepatitis B virus infection. In multivariate analysis, after adjusting for potential confounding factors, Women who were employed/student remained at reduced risk of being Hepatitis B virus infected (aOR 0.35, 95% CI 0.13–0.95).

**Table 3:** Association of positive HBsAg with clinico-demographic characteristics

Variable	cOR (95% CI)	*p*-value	aOR (95% CI)	*p*-value
Age in years	1.05 (0.97–1.14)	0.24	1.02 (0.93–1.13)	0.624
HIV	2.89 (1.08–7.73)	0.035	2.24 (0.77–6.48)	0.137
Married/Cohabiting	5.58( 0 .73–42.69)	0.098	4.66 (0.53–41.0)	0.165
Secondary/College education	0.39 (0.15–0.98)	0.046	0.41 (0.15–1.1)	0.076
Employed/Student	0.31 (0.12–0.78)	0.013	0.35 (0.13–0.95)	0.039

*cOR* crude odd ratio, *CI* confidence interval, *aOR* adjustable odd ratio

## Discussion

In the present study, the overall prevalence of HBsAg among pregnant women was 8.03%. In accordance to WHO interpretation, the finding grades the study setting an area with high HBV disease endemicity [16]. Our finding is in agreement with previous studies on HBsAg prevalence among pregnant women from high HBV endemic areas [17–19]. In comparison to other studies conducted in Tanzania, the prevalence of HBV among pregnant women in our study was higher than that reported in rural-southern (6.3%), northern (4.2%) and at national hospital (3.9%) [12, 20, 21]. On the other hand, the prevalence was lower than that reported previously among pregnant women in Kenya (9.3%), Uganda (11.8%) and Cameroon (10.2%) [22–24]. The observed variations could be attributable to geographical differences, since our study was performed in highly populated urban setting compared to previous studies in Tanzania and elsewhere.

In the current study, the prevalence of HBsAg was significantly high in pregnant women who had primary level of education compared to those with secondary/college level of education. Our observation was in agreement to a previous conducted in Nigeria [25]. Moreover, pregnant women who had no employment and those who were married or cohabiting had high prevalence of HBsAg. These findings are in contrast with previous results reported in Uganda (Married 10.6%), Ethiopia (low income 1.13%, married 2.64%) [23, 26]. It is not clear whether the differences observed from our study was due to high population of women who were unemployed and married/cohabiting or involvement in risky behavior in these group. Unemployment could fuel women to engage in risky sexual behavior including commercial sex, thus increasing risk of infection. Age of participants and gestation of the pregnancy were not significantly associated with high prevalence of HBsAg in line with other previous study in Ethiopia [27].

The overall prevalence of HIV among pregnant women in our study exceeded the recent reported national HIV prevalence among pregnant women [28]. Similarly, the prevalence was higher compared to results from previous studies among pregnant women in Tanzania [12, 29]. Likewise, the prevalence was higher than that reported in recent studies in Uganda (9.3%) and Ethiopia (6.6%), which are HIV endemic areas [23, 27]. The observed differences to this study maybe attributable to risk of sexual behavior since most of participants in the current study were less than 24 years, hence likely to be involved in high risky sexual behavior like prostitution. Likewise, this is the age were most participants may commence unprotected sexual interaction. In addition the study site has estimated population growth rate of 6.6% per year compared to national annual population growth rate of 2.9%, displaying high sexual activities in this setting [30], explaining the observed finding.

Prevalence of HBsAg was significantly high in pregnant women who were HIV positive compared to those who were HIV negative (*p* < 0.05). This finding is in contrast to HBsAg prevalence reported among HIV-positive and HIV-negative pregnant women at a tertiary hospital in Tanzania [12]. On the Contrary, studies from Sub-Saharan Africa have reported similar positivity rate of HBsAg prevalence among HIV-positive and HIV negative pregnant women [23, 31, 32]. The predominant mode of transmission is still thought to be childhood/perinatal in this country. The higher prevalence of HBV infection among HIV patients observed in our study could be due to those patients being less able to clear it.

The rate of HBV and HIV co-infection in our study was 2.8%. This rate was higher than that reported previously from a national hospital in Dar es Salaam, Tanzania [12]. Furthermore, the prevalence of HBV/HIV co-infection among pregnant women found in our study was higher than the previous reports in Nigeria (0.24%), Ethiopia (0.6%) and Cameroon (1.5%) [24, 33, 34]. However, the prevalence of HBsAg in the current study was lower than that reported from a recent study among pregnant women in South Africa (3.1%) [35]. The observed low HBV/HIV co-infection rates in settings of high HBV endemicity could be explained by the fact that the main routes of transmission of HBV in Sub-Saharan Africa could be vertical and likely occurs in early childhood.

Our study found that employment and being students were independent factors associated with reduced risk of HBV infection (aOR = 0.35; 95% CI 0.13–0.95). The finding of employment and students being protective to HBV infections was contrary to studies conducted earlier in Tanzania and elsewhere within the region [12, 27, 32]. Similar to other previous reports, marital status and education levels were not independently associated with HBV infections. HIV was also not independently associated with HBV infection, despite showing association in bivariate analysis. Similar finding was observed in previous studies among pregnant women in Malawi, Ethiopia and Cameroon [27, 32, 36]. In spite of lack of association between HBV and HIV in our study, prevalence of these infections remains high in Tanzania, as observed in the current study.

Our study had some limitations; detection of other HBV surrogate markers (HBeAg, HBc-antibodies, HBV DNA) and Hepatitis C virus were not possible due to financial constraints. We did not ascertain risk factors for HBV infection because of recall biases and design of the study. Despite that, our findings are based on a substantial sample size and the inclusion of municipal and health centers minimized the selection biases, since these are catchment settings where both uncomplicated and complicated cases are first seen.

## Conclusions

Our study findings suggest high prevalence of HBV and HIV infection among pregnant women in municipal health facilities in Dar es Salaam, Tanzania, while HBV/HIV co-infection is low. The results suggest that perinatal transmission is a common mode of HBV transmission in our setting. Based on our finding, we advocate integrating HBV infection into PMTCT program in Tanzania. Testing for HBsAg should be recommended for every pregnant women and possible prevention of mother-to-child transmission measures should be considered.

## Abbreviations

ALAT: Alanine aminotransferase
ANC: Antenatal clinic
aOR: Adjustable odd ratio
CI: Confidence interval
cOR: Crude odd ratio
DPT-HB: Diptheria, Pertusis, tetanus and Hepatitis B vaccine
HBcAg: Hepatitis B core antigen
HBeAg: Hepatitis B envelope antigen
HBsAg: Hepatitis B surface antigen
HBV: Hepatitis B virus
HIV: Human immunodeficiency virus
IQR: Interquartile range
PMTCT: Prevention of mother to child transmission
WHO: World Health Organization
